# Validation of the Kidney Donor Profile Index (KDPI) to assess a deceased donor’s kidneys’ outcome in a European cohort

**DOI:** 10.1038/s41598-019-47772-7

**Published:** 2019-08-02

**Authors:** Maximilian Dahmen, Felix Becker, Hermann Pavenstädt, Barbara Suwelack, Katharina Schütte-Nütgen, Stefan Reuter

**Affiliations:** 1 Department of Internal Medicine D, Division of General Internal Medicine, Nephrology and Rheumatology, University Hospital Münster, Münster, Germany; 2 Department of General and Visceral Surgery, University Hospital Münster, Münster, Germany

**Keywords:** Renal replacement therapy, Outcomes research

## Abstract

The Kidney Donor Profile Index (KDPI) was introduced in the United States in 2014 to guide the decision making of clinicians with respect to accepting or declining a donated kidney. To evaluate whether the KDPI can be applied to a European cohort, we retrospectively assessed 580 adult patients who underwent renal transplantation (brain-dead donors) between January 2007 and December 2014 at our center and compared their KDPIs with their short- and long-term outcomes. This led to the observation of two associations: one between the KDPI and the estimated glomerular filtration rate at one year (1-y-eGFR) and the other between the KDPI and the death-censored allograft survival rate (both *p* < 0.001). Following this, the individual input factors of the KDPI were analyzed to assess their potential to evaluate the quality of a donor organ. We found that a donor’s age alone is significantly predictive in terms of 1-y-eGFR and death-censored allograft survival (both *p* < 0.001). Therefore, a donor’s age may serve as a simple reference for future graft function. Furthermore, we found that an organ with a low KDPI or from a young donor has an improved graft survival rate whereas kidneys with a high KDPI or from an older donor yield an inferior performance, but they are still acceptable. Therefore, we would not encourage defining a distinct KDPI cut-off in the decision-making process of accepting or declining a kidney graft.

## Introduction

The global shortage of donor organs has led to increasing efforts to optimize the allocation process of renal allografts^1,2^.

While choosing a recipient, a critical issue araises in terms of evaluating the kidney graft quality. Several methods and scores have been employed to evaluate organ’s quality which mainly depend on clinical and, in part, histological donor data. However, some of these methods assess other recipient parameters as well^3–8^, as a histological examination can be limited in terms of availability, sampling errors, prolonged cold ischemia time (CIT), and inter-observer-variability^3^. Moreover, there is no evidence that histological assessment, may serve as an independent predictor of allograft survival unless there is readily accessible donor data^9–13^.

Furthermore, in the context of Eurotransplant, procurement biopsies are not routinely performed as the main focus is placed on the evidence-based objective of short ischemia times^14^.

During the mid-90’s, Chertow *et al*. identified advanced donor age as a leading determinant of reduced allograft survival^15^. Further studies have confirmed the validity of this finding and have associated it with the loss of functional nephrons with age^16,17^. Even in the standard criteria donor/extended criteria donor (SCD/ECD)-dichotomy, established in the United States (US) in 2002, age plays a decisive role as well^18^. Unfortunately, the ECD/SCD labeling has been proven to insufficiently predict the prognosis, as kidneys are suspected to be discarded unnecessarily due to the stigmatization that arises from the ECD/SCD label^19–21^.

At present, the Kidney Donor Profile Index (KDPI), based on the Kidney Donor Risk Index (KDRI), has been established as the most effective scoring system in the US, in terms of assessing the individual risk of a deceased donor’s kidney^22^.

The KDPI is a percentile measure that is based on the United Network of Organ Sharing’s (UNOS) data from The Scientific Registry of Trasplant Recipients (SRTR). It examines the risk of kidney failure for a given donor relative to the risk for an average kidney donor from the previous year. The KDPI has been implemented as the central component of a new kidney allocation system that was adopted by the US in 2014, which is basically a longevity matching allocation (the best kidneys –with the lowest KDPI– are allocated to the recipients with the longest predicted post-transplant survival rate). It replaced the former SCD/ECD-dichotomy to optimize the utilization of kidneys and prohibit unnecessary discards^22,23^.

Calculation of the KDPI includes ten variably weighted donor parameters that significantly influence donors’ organ quality with particular focus on nephron mass: age, height, weight, cause of death, last serum creatinine, history of diabetes, hypertension, HCV-infection, ethnicity, and the discrimination between donation after brain death versus donation after cardiac death^22^.

Of all the variables listed above, age has the highest negative impact in the KDPI algorithm, especially if a donor is above 50 years or below 18 years. Furthermore, the KDPI increases, when the donor is shorter and lighter than 80 kg or shows an increased serum creatinine level.

In Germany there is no valid stratification score or tool to assist physicians in their decision of accepting or declining an organ offer. However, as the data required to calculate the KDPI is available in Germany, we evaluated the KDPI using retrospective data from our transplant center. The results were interpreted by taking into account that the Eurotransplant allocation system is fundamentally different from the US one and that German conditions with respect to health care system, population, and geographics differ from those in the US.

## Patients and Methods

### Study design, setting, and participants

We conducted a retrospective single-center cohort study of 580 adult patients who received a deceased donor renal transplantation (RTx) at the University Hospital of Münster, Germany between January 2007 and December 2014. Recipients younger than 18 years as well as living donor recipients were excluded (see Supplementary Fig. S1).

Prior to analysis, the data of all patients were anonymized. The study was approved by the local ethics committee (Ethik Kommission der Ärztekammer Westfalen-Lippe und der Medizinischen Fakultät der Westfälischen Wilhelms-Universität, 2014-381-f-N), and the methods adopted by this study were conducted in accordance with the current transplantation guidelines and the Declarations of Istanbul and Helsinki. Furthermore, written informed consent was obtained from all participants at the time of transplantation permitting recording and using their clinical data for scientific research. As donors were braindead at the time of donation and as this study is a retrospective, non-interventional study, written informed consent of donors was waived. Organ donors had previously expressed a wish to donate their organs in legal terms. No samples were procured from prisoners. Organs were procured on behalf of Eurotransplant and the Deutsche Stiftung Organtransplantation (DSO) in different hospitals of the Eurotransplant area. Transplantations were performed by the Department of General, Visceral and Transplantation Surgery at the University Clinics Münster, Germany.

To display the unexceptional transplant population and contracted by the limitations of retrospective considerations, no further inclusion- or exclusion criteria were implemented.

### Data sources

Donor data was obtained from the Eurotransplant Network Information System (ENIS) and the KDRI and KDPI were assessed using the Organ Procurement and Transplantation Network (OPTN) online calculator^22^.

In case of missing information on the history of diabetes or hypertension data was substituted by mean values according to the given algorithm (approximately 31% and 9% for hypertension and diabetes, respectively). Recipient and outcome parameters at baseline and during follow-up were retrospectively collected from the patients’ files. The recipients were regularly revisited within the scheduled out-patient aftercare and treated in-patient in case of complications.

### Confounding parameters

The set of covariables used in our study is based on theoretical considerations. We selected common parameters that have been established by numerous studies over a long time period which investigated outcome and risk factors in renal transplantation^24^.

These confounders have been chosen to cover the fundamental categories of outcome related pre- and peritransplant parameters that are not included in the KDPI: basically physical and medical recipient characteristics, procedural aspects and immunological conditions. However, the number of included variables has to be limited to a number which is appropriate for our sample size.

### Outcome measures

Primary outcome measure was the estimated glomerular filtration rate (eGFR) at one year after transplantation (1-y-eGFR) pertaining to patients alive at this point of time, as the quantitative graft function at one year has been established as an appropriate indicator for long term transplant success^25–27^.

The eGFR was calculated using the CKD-EPI formula and was based on a whole-blood analysis for creatinine (enzymatic assy; Creatinine-Pap, Roche Diagnostics, Mannheim, Germany).

Patient survival was defined as the time from RTx to death (from any cause) or last contact with alive patients. Death-censored graft survival was defined as the time from RTx to graft failure where death without prior graft failure was regarded as censored.

Secondarily, we assessed the incidences of delayed graft function (DGF), defined as need of dialysis within the first week post-transplantation, frequencies of biopsy-proven acute rejections (AR) within the first year and rates of complications requiring surgical intervention, also within the first year.

Following a preliminary series of univariate analyses for the chosen set of potential covariates (see Supplementary Tables S1 and S2), we conducted two models of multivariable analyses, first checking the KDPI’s influence on the chosen outcome measures (Model 1) and then investigating the impact of its individual input factors in the second step (Model 2).

Additionally, the KDPI was divided into three categories, as suggested by the OPTN: <35%, 35–85% and >85%^22^. Donor age was classified into three categories as well: <40 years, 40–60 years and >60 years – of which the upper cut-off conforms to the older ECD-criteria. These ranges were chosen to match with the mean donor ages of the above-named KDPI-categories.

### Statistical methods

Statistical analysis was performed using IBM SPSS® Statistics 25 for Windows (IBM Corporation, Somers, NY, USA). Continuous variables are shown as the median ± interquartile range (IQR) and the absolute and relative frequencies were provided for categorical variables.

Multivariable linear regression was conducted to determine independent factors that influence the 1-y-eGFR. We included the following recipient- and procedure-specific variables which, according to the aforementioned preconsiderations and preliminary univariate analyses, can potentially impact on the graft’s function: age, sex, body mass index (BMI), dialysis vintage (DV), CIT of the donor organ, condition of previous RTx, human leucocyte antigen (HLA) -matching and current panel reactive antibodies (cPRA%).The results were presented as regression coefficients (B) with 95%-confidence intervals.

To estimate the probability of DGF, acute rejections, and surgical complications multivariable binary logistic regression analyses were conducted. Therefore, a multivariable model was built using a stepwise variable selection procedure (inclusion: p-value of the score test ≤0.05, exclusion: p-value of a likelihood ratio test >0.1). The variables included age, sex, BMI, time on dialysis, CIT, condition of previous RTx, HLA-matching, and cPRA%. The results were presented as odds ratios (OR) with 95% confidence interval (95% CI) and p-value of a likelihood-ratio test. For the non-selected variables in the multivariable analyses, the p-value of the score test was provided.

Patient and death-censored allograft survival (based on the assumption that death is unrelated to the transplant) were estimated using the Kaplan-Meier method^28^, and KDPI- and donor’s age-groups were compared using log-rank tests. Cox proportional hazards regression models^29^ were built using a stepwise variable selection procedure to assess the association between the KDPI and patient and allograft survival while simultaneously adjusting for potential confounding factors (inclusion: p-value of the score test ≤0.05, exclusion: p-value of the likelihood ratio test >0.1). The results were presented as hazard ratios (HR) with 95% confidence interval (95% CI) and the p-value ofa likelihood-ratio test. For the non-selected variables in the multivariable analyses, p-value of the score test is given.

Analogous analyses were also conducted by including the aforementioned recipient- and procedure-specific variables, but instead of considering the fully computed KDPI each of its input parameter (respectively those that were variable within our cohort, such as donor age, BMI, the last serum creatinine before transplantation, history of diabetes or hypertension, and the cause of death) were taken as individual variables to examine their discrete predictive value with respect to the examined outcomes (Model 2), which were again adjusted to the same selection of covariates.

The correlation between the KDPI and donor age was investigated by assessing Pearson’s and Spearman’s coefficients of correlation.

No adjustments were made for multiple testing and the analyses were regarded as explorative. P-values < 0.05 were considered as statistically noticeable.

## Results

### Descriptive data

Tables 1 and 2 put forth the descriptive statistics of recipient- and procedure-specific characteristics as well as donor characteristics for the total patient collective and for the three groups of the KDPI ranges.

**Table 1:** Baseline recipient characteristics sorted by KDPI ranges (no = number; med. = median; IQR = interquartile range; DV = dialysis vintage in month, CIT = cold ischemia time in hours, HLAmm = HLA-mismatches).

Recipient- and procedure-specifics Parameter	N	All	N	KDPI < 35%	N	KDPI 35–85%	N	KDPI > 85%
Age, years, med. (IQR)	580	57.4 (19.9)	78	50.6 (15.6)	309	51.5 (17.3)	191	68.3 (6.2)
Men, no (%)	580	350 (60.3)	78	46 (59.0)	309	176 (57.0)	191	127 (66.5)
BMI, kg/m^2^, med. (IQR)	580	25.3 (5.8)	78	24.0 (5.5)	309	25.2 (5.9)	191	25.8 (5.6)
DV, mo, med. (IQR)	580	72.2 (56.4)	78	89.5 (54.9)	309	81.7 (49.0)	191	52.4 (41.7)
CIT, h, med. (IQR)	579	10.0 (6.0)	78	12.1 (6.4)	309	10.7 (5.6)	190	9.8 (4.3)
PreviousRTx, no (%)	580	64 (11.0)	78	12 (15.4)	309	39 (12.6)	191	13 (6.8)
0–3 HLAmm., no (%)	577	399 (68.8)	78	57 (73.1)	307	258 (83.5)	190	83 (43.5)
4–6 HLAmm., no (%)	577	181 (31.2)	78	21 (26.9)	307	51 (16.5)	190	108 (56.5)
cPRA, %, med. (IQR)	577	0.0 (0.0)	78	0.0 (0.0)	307	0.0 (0.0)	190	0.00 (0.0)

**Table 2:** Baseline donor characteristics sorted by KDPI ranges (no = number; med. = median; IQR = interquartile range; sCrea = donor’s lat serum-creatinine, cerebrovasc. = cerebrovascular cause of death, CNS = central nervous system).

Donor-Parameter	N	All	N	KDPI < 35%	N	KDPI 35–85%	N	KDPI > 85%
Age, years, med. (IQR)	580	55.0 (21.0)	78	29.5 (13.3)	309	51.0 (11.0)	191	71.0 (9.0)
Men, no (%)	580	278 (47.9)	78	44 (56.4)	309	154 (49.8)	191	78 (40.8)
BMI, kg/m², med. (IQR)	578	26. 2 (5.4)	78	24.3 (4.5)	309	26.8 (5.2)	191	26.3 (4.0)
sCrea, mg/dl, med (IQR)	578	0.85 (0.55)	78	0.77 (0.4)	309	0.85 (0.55)	191	0.91 (0.70)
**History of Diabetes**								
- confirmed no (%)	578	59 (10.2)	78	4 (4.9)	309	15 (4.9)	191	41 (21.5)
- unknown, no (%)		409 (70.5)		54 (65.9)		229 (74.1)		126 (66.0)
**History of Hypertension**								
- confirmed, no (%)	578	182 (31.4)	78	0	309	88 (28.5)	191	94 (49.2)
- unknown, no (%)		324 (55.9)		55 (70.5)		181 (58.6)		87 (45.5)
**Cause of Death**								
- Anoxia, no (%)	578	79 (13.6)	78	11 (14.1)	309	50 (16.2)	191	18 (9.4)
- Cerebrovasc., no (%)		375 (64.7)		23 (29.5)		209 (67.6)		143 (74.9)
- Head Trauma, no (%)		89 (15.3)		33 (42.3)		31 (10)		25 (13.1)
- CNS-Tumor, no (%)		4 (0.7)		1 (1.3)		2 (0.6)		1 (0.5)
- Other, no (%)		31 (5.3)		10 (12.8)		17 (5.5)		4 (2.1)

Additionally all donors were white, HCV-negative and did not meet DCD-criteria.

**Figure 1:**
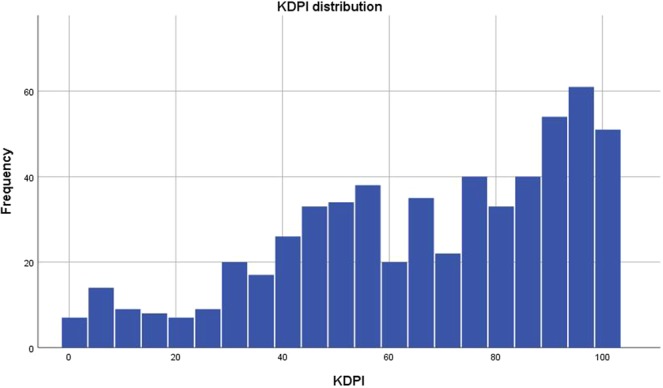
KDPI distribution across the examined cohort.

In our cohort of 580 kidney transplant recipients the median KDPI was 71% (IQR: 47–91%) (Fig. 1), and the mean age of both donors and recipients was 55 years. However, while 60% of the recipients were male, the donors were equally distributed in terms of sex.

CIT averaged at 10 hours and 11% of all patients underwent previous RTx. The mean dialysis vintage was 72 months, and interestingly, lower KDPIs were associated with longer waiting times (<35% KDPI kidney: 89.5 ± 54.9 months versus 52.4 ± 41.7 months for >85% KDPI).

The KDPI increased in parallel with the donors’ last creatinine level. Ten percent of donors were diabetics; approximately one third had a history of hypertension and two-thirds died due to a cerebrovascular event.

### Renal function

**Figure 2:**
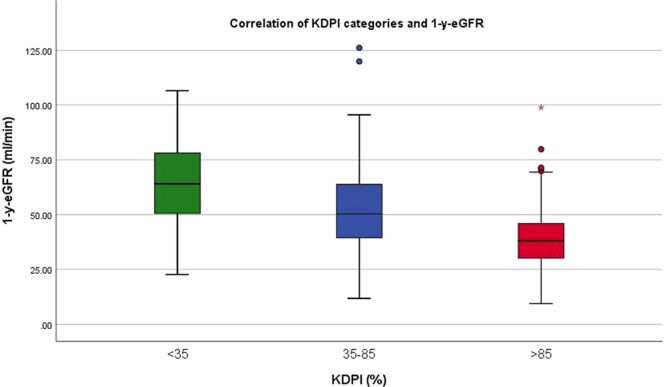
Means of 1-y-eGFR for KDPI categories. 1-y-eGFR decreases with higher KDPI.

**Figure 3:**
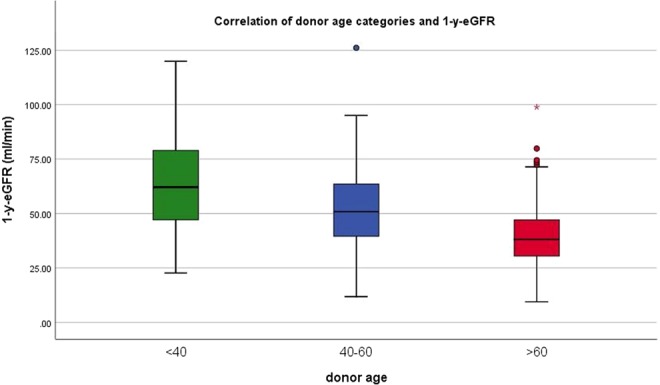
Means of 1-y-eGFR for donor age categories. 1-y-eGFR decreases with higher donor age.

The mean 1-y-eGFR was found to be 50 ± 20 ml/min/1.73 m^2^. Furthermore, the 1-y-eGFR was found to decrease as the KDPI range increased (65 ± 19, 53 ± 19, 39 ± 15 ml/min/1.73 m^2^ for KDPI < 35%, 35–85% and >85%, respectively, Fig. 2). A similar distribution of 1y-eGFR means was found while conducting an analogous analysis for donor age categories (Fig. 3), reflecting a proper correlation between the selected donor age categories and the mean donor age of the chosen KDPI categories.The differences were marginal and both correlations proved to be equally significant. Only direct comparison reveals a faint superiority of the KDPI (see Supplementary Table S4).

The multivariable linear regression analysis that was adjusted to include recipient characteristics (Model 1) revealed a noticeable association between KDPI and 1-y-eGFR. Further variables that were associated with 1-y-eGFR included a higher recipient BMI, a longer CIT and a higher number of HLA-mismatches. The analogous analysis was conducted after adjustments for each KDPI input parameter (Model 2). Here, we found donor age to significantly influence 1-y-eGFR. Additional donor factors that correlated with 1-y-eGFR included the last serum creatinine and a cerebrovascular cause of death – in fact, these were two of the three ECD-criteria (Table 3).

**Table 3:** Multivariate analysis of predictors of theeGFR one year after RTx in ml/min/1.73 m^2^ using stepwise forward selection in linear regression analysis (for details, see Methods).

Primary Outcome 1-y-eGFR (Linear Regression)	Model 1		Model 2	
Independent variable	B (95% CI)	*P*	B (95% CI)	*P*
KDPI, %	−0.338 (−0.396–−0.280)	<0.001	—	<0.001
Donor Age, years	—		−0.478 (−0.574–−0.381)	0.006
Donor Sex (male)	N/S: 0.099		−4.320 (−7.388–−1.251)	
Recipient Age, years	N/S: 0.401		N/S: 0.549	
Recipient Sex (male)	N/S: 0.145		N/S: 0.255	
Recipient BMI, kg/m²	−0.759 (−1.1135–−0.383)	<0.001	−0.797 (−1.165–−0.428)	<0.001
Dialysis Vintage, months	N/S: 0.667		N/S: 0.900	
Cold Ischemia Time, hours	N/S: 0.073		N/S: 0.057	
Previous RTx	N/S: 0.607		N/S: 0.466	
Current PRA%	N/S: 0.369		N/S: 0.259	
>3 HLA-mismatches	N/S: 0.177		N/S: 0.280	
Donor Last Serum-Creatinine	—		−3.980 (−6.294–−1.666)	0.001
Donor BMI, kg/m²	—		N/S: 0.206	
Donor Hypertension	—		N/S: 0.362	
Donor Diabetes mellitus	—		N/S: 0.978	
Donor Death by Anoxia	—		N/S: 0.232	
Donor cerebrovascular Death	—		−5.792 (−9.137–−2.446)	0.001
Donor Death by Head Trauma	—		N/S: 0.213	
Donor Death by CNS-Tumor	—		N/S: 0.251	
Donor Death by other cause	—		N/S: 0.768	

Given are coefficients of regression (B) with 95% confidence interval (95% CI) and *p*-value of a likelihood-ratio test for the selected variables. For non-selected (N/S) variable *p*-value of the score test is displayed.

### Patient and allograft survival

For the total time of follow-up 63 patients suffered from total graft failure (11%) and 47 died with functioning graft (8.1%). 13 (2.2%) patients died after graft failure due to other reasons.

**Figure 4:**
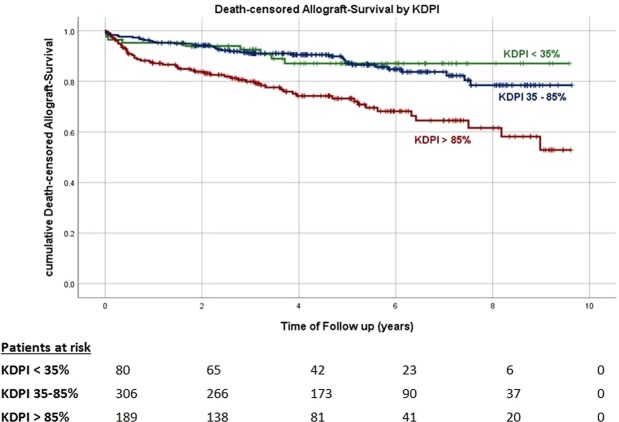
Kaplan-Meier curves for death-censored allograft survival by KDPI-categories. p- values of log-rank-Test: <35% vs 35–85% p = 0.116. 35–85% vs >85% p = 0.003. <35% vs >85% p < 0.001.

**Figure 5:**
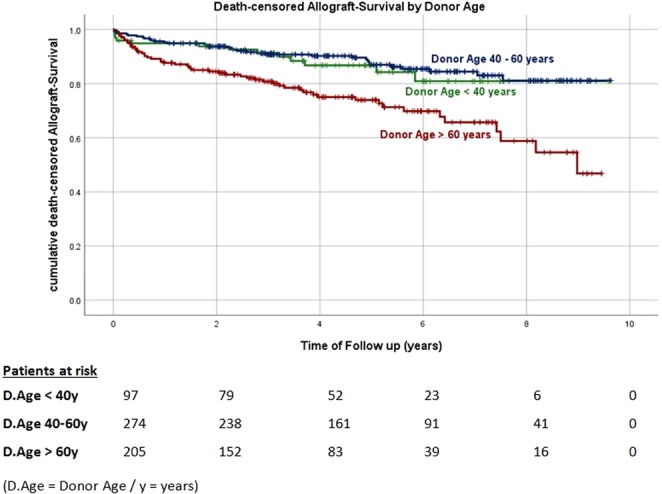
Kaplan-Meier curves for death-censored allograft survival by donor age categories. p-values of log-rank-Test: <40 vs 40–60 years p = 0.194. 40–60 vs >60 years p < 0.001. <40 vs >60years p = 0.011.

Kaplan-Meier curves for death-censored allograft survival by KDPI category are shown in Fig. 4. Allograft survival was noticeably reduced in recipients of kidneys with a KDPI > 85% compared to recipients of kidneys with a lower KDPI (<35% vs. >85%, log-rank p < 0.001 and 35–85% vs. >85% long-rank p = 0.003). Similar results were found when allograft survival was analyzed according to the donor age category (Fig. 5). Kidneys in a higher donor age category showed a noticeable decrease in allograft survival rate compared to kidneys from younger donors (<40 years vs. >60 years log-rank p = 0.011 and 40–60 years vs. > 60 years log-rank p < 0.001). Cox-regression adjusted to the aforementioned selection of potential confounders revealed a noticeable association between the KDPI and allograft survival HR 1.026 (95%-CI 1.013–1.039), p < 0.001, Model 1, Table 4. Analogously, we found a higher donor age to be independently associated with allograft survival HR 1.037 (95%-CI 1.018–1.057), p < 0.001, Model 2, Table 4.

**Table 4:** Multivariate analysis of predictors of death-censored allograft survivalusing stepwise forward selection in Cox regression analysis (for details, see Methods).

Death-censored Allograft-Survival (Cox Regression)	Model 1		Model 2	
Independent variable	HR (95% CI)	*P*	HR (95% CI)	*P*
KDPI, %	1.026 (1.013–1.039)	<0.001	—	
Donor Age, years	—		1.037 (1.018–1.057)	<0.001
Donor Sex (male)	N/S: 0.155		N/S: 0.089	
Recipient Age, years	N/S: 0.845		N/S: 0.604	
Recipient Sex (male)	N/S: 0.723		N/S: 0.750	
Recipient BMI, kg/m²	N/S: 0.570		N/S: 0.578	
Dialysis Vintage, months	N/S: 0.455		N/S: 0.561	
Cold Ischemia Time, hours	1.066 (1.009–1.125)	0.021	1.060 (1.005–1.118)	0.032
Previous RTx	N/S: 0.346		N/S: 0.484	
Current PRA%	N/S: 0.752		N/S: 0.653	
>3 HLA-mismatches	N/S: 0.927		N/S: 0.785	
Donor Last Serum-Creatinine	—		N/S: 0.488	
Donor BMI, kg/m²	—		N/S: 0.541	
Donor Hypertension	—		N/S: 0.747	
Donor Diabetes mellitus	—		N/S: 0.732	
Donor Death by Anoxia	—		N/S: 0.981	
Donor cerebrovascular Death	—		N/S: 0.274	
Donor Death by Head Trauma	—		N/S: 0.172	
Donor Death by CNS-Tumor	—		N/S: 0.564	
Donor Death by other cause	—		N/S: 0.930	

Given are hazard ratios (HR) with 95% confidenceinterval (95% CI) and p-value of a likelihood-ratio test for the selected variables. For non-selected (N/S) variable p-value of the score test is displayed.

No difference was found between the groups in terms of patient survival, neither when analyzed by KDPI nor by donor age category (see Supplementary Table S3).

### Secondary outcome parameters

Neither the KDPI nor donor age was found to significantly influence the secondary outcome parameters. Frequencies of DGF were similar among the three KDPI categories.

The multivariable logistic regression analysis revealed that the recipient’s BMI, CIT, and a previous RTx had an influence on the incidence of DGF in Model 1 and 2 highlighting the importance of considering and minimizing these additional risk factors whenever possible. In Model 2, dialysis vintage and the donor’s last creatinine level were also found to have a noticeable impact on the incidence of DGF (Table 5).

**Table 5:** Multivariate analysis of predictors of further secondary outcomes using stepwise forward selection in binary logistic regression.

Secondary Outcomes (Binary Logistic Regression)		Model 1		Model 2	
Outcome	Significant Parameter	Odds Ratio	*P*	Odds Ratio	*P*
Delayed Graft Function (within 1 week after RTx)	Recipient BMI, kg/m²	1.062	0.013	1.064	0.011
	Previous RTx	0.477	0.015	0.452	0.008
	CIT, hours	1.056	0.022	1.065	0.010
	Donor Sex (male)	1.572	0.026	—	—
	Dialysis Vintage	—	—	1.006	0.028
	Donor Last Creatinie	—	—	1.686	<0.001
Acute Rejections within 1 year after RTx	Recipient Sex (male)	1.869	0.011	1.869	0.011
Surgical Complications within 1 year after RTx	Recipient Age, years	1.021	0.007	1.021	0.007
	Donor Sex (male)	1.498	0.029	1.498	0.029
	CIT, hours	1.053	0.020	1.053	0.020

Given are Odds Ratios and p-value of a likelihood-ratio test for selected variables. Non-selected (N/S) variables are not displayed.

Male recipients were more frequently affected by acute rejection than female recipients during the first year post-RTx in both models.

Surgical complications were found to occurre more frequently in older male recipients as well as in recipients of male donors and of kidneys with a longer CIT.

### Correlations of parameters/scores

**Figure 6:**
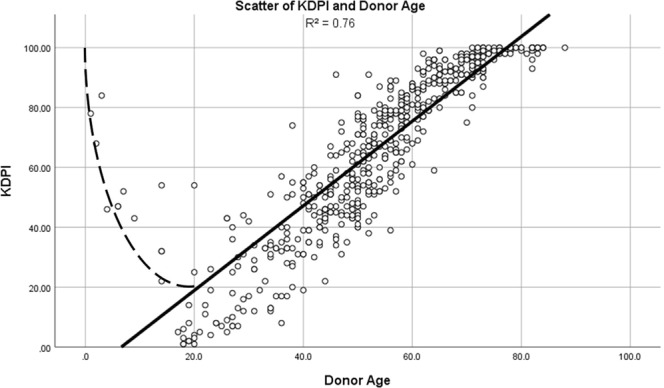
Scatter of KDPI and donor age. Scatter-plot illustrating the correlation of KDPI and donor age.

Both examined predictive parameters, KDPI and donor age have been found to be directly correlated to each other (Fig. 6: R² = 0.76).

Discrepancy largely arises due to the KDPIs algorithm, as explained earlier. A donor who is below the age of 18 or of an advanced age increases the calculated value, resulting in the outliers on the left hand side of Fig. 6. This effect is attenuated when only adult donors are included (R² = 0.85). The correlation coefficient is 0.929 in Spearman’s rank (p < 0.001) and 0.870 in Pearson’s rank (p < 0.001), indicating that an increase in the index is predominantly driven by an increase in donors’ age in our cohort.

Concerning the primary outcome of 1-y-eGFR we conducted a third model of linear regression analysis including the KDPI (as the leading parameter of Model 1) and the donor age (as the leading parameter of Model 2) but no other covariates to create an unbiased comparison. Both parameters were entered into the analysis without applying any method of selection. Herein the KDPI (p < 0.001) was found to be marginally superior to donor age (p 0.004) (see Supplementary Table S4).

Additonally, receiver-operating-characteristic (ROC) -analyses were conducted to evaluate the performance of the KDPI, donor age, and ECD/SCD in terms of predicting allograft failure (see Supplementary Fig. S2). The KDPI and donor age resulted in similar moderate but highly significant predictive values, whereas a true ECD-criterion cannot significantly predict graft failure (see Supplementary Tables S5 and S6).

## Discussion

The optimal allocation of donated organs is one of the major challenges in transplant medicine. Clinicans who decide whether to accept or decline a kidney offer walk a tightrope in weighing a potentially limited graft survival rate with consecutive need for dialysis treatment with the undefined waiting time for the next organ offer, which does not guarantee good quality or a perfect matching^30^.

The KDPI, which can be calculated by an easily accessible online tool, was established to objectify and facilitate the decision-making process for transplant physicians. Its primary purpose is to objectively implement “longevity matching” allocation, aiming to allocate the best kidneys to those recipients with the best long-term prognosis.

The US data demonstrated that a deceased donor kidney with a KDPI lower than 20% nearly reaches the half-life of a living donation (11.4 vs. 12 years), whereas a kidney with a KDPI > 85% does not accomplish half of this period^1^.

However, the question remains whether this discrepancy arises due to the fact that “inferior” organs are selected for “sicker” patients.

Several studies have been conducted among American and European populations to evaluate the validity of the KDPI to determine a dontation’s quality^9,31–33^.

A Canadian paper from 2017 has highlighted the need to determine the applicability of KDPI in different regions, as its application did not seem to improve the prediction of allograft failure in a British Columbian cohort in comparison with the simple criterion of donor age^34^. Lee and Abramowicz from Antwerp in Belgium anticipated the KDPI in 2015 to become an advantageous tool to qualitatively evaluate kidney donations across Europe^3^. After completing their own investigations in 2017, they confirmed that the KDPI provides good guidance regarding whether to accept or decline an organ offer, and they encourage other European centers to validate this score^35^. The most recent studies in the field were conducted in Berlin, which demonstrated the utility of the KDPI in terms of donor quality assessment in a German cohort^36^, and from Barcelona. Both studies still demand more data before suggesting the use of the KDPI in clinics^37^. The present study confirms the applicability of the KDPI in a kidney transplant cohort of Münster, Germany, showing it has good predictive power in terms of 1-y-eGFR and death-censored allograft survival. Our median KDPI matches quite well with those from Berlin^36^ and Barcelona^37^ and thus appears to represent the European donor population.

We notably found that an improved graft survival of an organ with a low KDPI is contrasted by a longer waiting time (which is usually associated with reduced overall survival). One explanation for this might be that older organs (with higher KDPI) are often allocated by the Eurotransplant algorithm of the European Senior Programm (ESP), which aims to shorten waiting time for patients older than 65 years. In this regard, we observed an impaired but still acceptable outcome of kidneys with a KDPI > 85%. The eGFR was approximately 10 ml/min below total average (40 vs. 50 ml/min), but total graft failure was observed in only 13.7% vs. 8.6% of cases. As the waiting time for these organs was found to be 16 months less on average, we would advise transplant physicians to not generally decline an organ donor just because of a KDPI > 85%.

This result is consistent with the results of Lehner *et al*. who found significantly worse but acceptable outcomes of high and even very high KDPI kidneys as well (10-year-death-censored graft survival of 62% for KDPI > 85%) and also emphasized the benefits of the ESP program in this context^36^.

In summary, the KDPI may be a supportive tool in objectifiying clinical evaluations and ranking the prognosis to some extent in clinican’s decision-making process.

The general predictive power of the KDPI is moderate. In the original trial the c-statistics was 0.6, which means that outcomes for organs with strongly different KDPI values can be prognosed more precisely than for organs with similar KDPI values^22^. Therefore, according to our data the use of a distinct KDPI cut-off cannot be recommended.

The correlation between donor age alone and our leading outcomes was highly significant as well, which is not surprising, given that both parameters were closely correlated.

The KDPI may be marginally superior, as it takes creatinine level and cerebrovascular cause of death into account, which prove to be of additional significance concerning our primary outcome measure. This assumption is supported by the results of the aforementioned bivariate linear regression analysis shown in the Supplementary Table S4 and the receiver operating characteristics presented in the Supplementary Table S5 and Fig. S2.

With respect to the other KDPI input factors, we did not observe them to have any relevant impact on the primary outcome with three of them not even showing any variation within the examined cohort. As illustrated in Fig. 6, high KDPI socres appeared to be predominantly caused by donor’s age, however, this association was found to be noticeably lower in the more heterogenous population of the US, probably due to socio-demographic- and health-care-related reasons. In particular, characteristics that are independent of age, such as ethnicity and HCV infection did not vary within our cohort. Therefore, a US-derived scoring system will probably remain suboptimal under European conditions.

However, the KDPI might provide certain advantages compared to the SCD/ECD labeling (Supplementary Fig. S2), not only in terms of avoiding a dichotomy but also in terms of processing the given information more precisely. Though a dichotomous tool (like the SCD/ECD label) seems to facilitate clinical decision making more than a continuous scale (like the KDPI), several studies have demonstrated the insufficient performance of ECD/SCD which was related e.g. to oversimplification. Especially, the validation of the ECD criteria in kidney transplant centers outside of the US and its correlation with outcomes is poor. Moreover, the KDPI is a continuous scale-based algorithm which incorporates more donor characteristics (10 for KDPI vs. 4 for ECD)^19–21^. In the SCD/ECD algorithm creatinine level, hypertension, and death by stroke are only relevant within the short period between 50 and 59 years. Moreover, this algorithm disregards the combination of negative predictors It has been proven that the outcome of SCD-kidneys with high KDPI scores is comparable to ECD-kidneys with a medium KDPI^35^.

The spectrum of age and comorbidities of donors as well as of the recipients has increased over the time. Therefore, a more sophisticated score has been demanded, guiding the clinicians’ decision making especially in complex and marginal cases to enable still an optimized allocation and utilization. To this end, the KDPI provides an additional ranking within the important group of equivalently ECD-labeled kidneys and allows to set individual thresholds for distinct patients. In our study, the KDPI significantly predicts 1-y-eGFR (p < 0,001) and death-censored graft failure (p = 0.01) when selecting only ECD-kidneys for analogous multivariable analyses (n = 274). Therefore, we can state at least, that the KDPI provides valid and differentiated predictive information, where the ECD-criterion is just equivalently true.

Another advantage of the KDPI is that it absorbs donor characteristics that are at risk of becoming (according to our data) unjustifiably important when considered separately.

However, a disadvantage of the KDPI might be that it could generate a labeling effect a fortiori. Whereas a simply ECD-labeled kidney was considered to be generally usable for selected and consented patients, a high KDPI is supposed to be more discouraging. Certainly, further prospective studies have to evaluate this issue.

Actually, within the Eurotransplant region the SCD/ECD rating would have no applicability as it would interfere with the Eurotransplant Senior Programm. In contrast the KDPI is applicable in all allocation subsystems. However, the application of the KDPI should be evaluated in a prospective study. Our retrospective approach can only be hypothesis generating.

Our finding of an additional adverse effect of the recipient’s BMI is supported by further studies^38,39^, although one of the latest reviews describes this effect to be only “slight”^40^.

We have to point out that a potential impact of the underlying renal disease, differences in the immunosuppressive therapy, infectious complications as well as anatomically, traumatically and procedurally caused graft anomalies were not considered within this study.

Furthermore, this study does not contain information about the kidneys that were discarded during the follow-up period, especially information regarding their KDPI and age, the reason for discarding them and the outcome of their potential recipients. Thus, further studies need to be conducted to provide an in-depth clarification about the fate of these kidneys.

The data collected for this study were analyzed retrospectively, and all inferential statistics were interpreted in an exploratory manner. It should be noted that potential errors might have been introduced, as they are inherent when maintaining a single-center database.

From our data, we conclude that the KDPI can be applied to approximately predict the short-and long-term functional outcomes of RTx in a European cohort.

Furthermore, our results suggest focussing on donor age as the most significant input parameter of the KDPI, as it can substantially determine the KDPI under German conditions. Presumably, the general predictive power of age is barely lower than that of the KDPI but age may serve as a simple rule of thumb to estimate the graft’s prognosis. In difficult cases it may be reasonable to calculate the KDPI and include additional information such as the patients’ BMI and the HLA-matching.

Propectively, we suggest establishing an adjusted KDPI score specific to European cohorts. This score should assist transplant physicians and paitients in deciding whether to accept or decline an organ offer.

## Supplementary information

Supplementary Information
