# Supplementary material for: Validation of the Kidney Donor Profile Index (KDPI) to assess a deceased donor’s kidneys’ outcome in a European cohort
Source: Sci Rep. 2019 Aug 2;9:11234. doi: 10.1038/s41598-019-47772-7 (PMC6677881; doi:10.1038/s41598-019-47772-7)
Supplement: Supplementary file 1 — Supplementary Information [file 41598_2019_47772_MOESM1_ESM.docx]

**Validation of the Kidney Donor Profile Index (KDPI) to assess a deceased donor’s kidneys’ outcome in a European cohort**

Maximilian Dahmen^1^, Felix Becker^2^, Hermann Pavenstädt^1^, Barbara Suwelack^1^, Katharina Schütte-Nütgen^1#^, Stefan Reuter^1#^*

^1^ Department of Internal Medicine D, Division of General Internal Medicine, Nephrology and Rheumatology, University Hospital Münster, Münster, Germany

^2^ Department of General and Visceral Surgery, University Hospital Münster, Münster, Germany

^#^ equally contributed

*Corresponding author

**Stefan Reuter**, Department of Medicine D, University Hospital of Muenster,

48149 Münster, Germany. [sreuter@uni-muenster.de](mailto:sreuter@uni-muenster.de)

Telephone: +49-251-83-47540

Fax: +49-251-83-56973

# Supplementary Information

## Supplementary Table S1

| **Univariate Analyses** | **Primary Outcome 1-y-eGFR**  **(Linear Regression)** | **Death-censored Allograft-Failure**  **(Cox Regression)** |
| --- | --- | --- |
| **Independent variable** | **B (95% CI) *P*** | **HR (95% CI) *P*** |
| KDPI, % | -0.348 (-0.407 – -0.290) <0.001 | 1.023 (1.010 – 1.037) 0.001 |
| Donor Age, years | -0.553 (-0.648 - -0.458) <0.001 | 1.031 (1.011 – 1.051) 0.003 |
| Donor Sex (male) | -0.445 (-7.914 - -0.976) 0.012 | N/S: 0.103 |
| Recipient Age, years | -0.409 (-0.544 – 0.273) <0.001 | N/S: 0.207 |
| Recipient Sex (male) | N/S: 0.083 | N/S: 0.314 |
| Recipient BMI, kg/m² | -0.983 (-1.401 – 0.565) <0.001 | N/S: 0.370 |
| Dialysis Vintage, months | 0.061 (0.015 – 0.106) 0.009 | N/S: 0.077 |
| Cold Ischemia Time, hours | N/S: 0.165 | N/S: 0.540 |
| Previous RTx | 6.117 (0.610 – 11.624) 0.030 | N/S: 0.889 |
| Current PRA% | N/S: 0.258 | N/S: 0.280 |
| >3 HLA-mismatches | -7.127 (-10.920 – -3.333) <0.001 | N/S: 0.601 |
| **Table S1:** Univariate analyses of predictors of the eGFR one year after RTx in ml/min/1.73m^2^ using linear regression analysis and of death-censored allograft failure using Cox regression. Given are coefficients of regression (B), and hazard ratio (HR) with 95% confidence intervals (95% CI) and the *p*-value of the likelihood-ratio test for selected variables. For non-selected (N/S) variable the p-value of the score test is displayed. | | |

## Supplementary Table S2

| **Univariate Analyses** | **Delayed Graft Function**  **(within 1 week after RTx)** | **Acute Rejections**  **within 1 year after RTx** | **Surgical Complications**  **within 1 year after RTx** |
| --- | --- | --- | --- |
| **Independent variable** | **OR *P*** | **OR *P*** | **OR *P*** |
| KDPI, % | N/S: 0.350 | N/S: 0.285 | N/S: 0.054 |
| Donor Age, years | N/S: 0.914 | N/S: 0.968 | 1.012 0.033 |
| Donor Sex (male) | 0.602 0.011 | N/S: 0.169 | 0.640 0.014 |
| Recipient Age, years | N/S: 0.566 | N/S: 0.424 | 1.017 0.018 |
| Recipient Sex (male) | N/S: 0.278 | 0.559 0.016 | N/S: 0.684 |
| Recipient BMI, kg/m² | N/S: 0.072 | N/S: 0.367 | N/S: 0.531 |
| Dialysis Vintage, months | N/S: 0.105 | N/S: 0.197 | N/S: 0.826 |
| Cold Ischemia Time, hours | 1.061 0.011 | N/S: 0.874 | 1.045 0.039 |
| Previous RTx | 1.866 0.029 | N/S: 0.261 | N/S: 0.458 |
| Current PRA% | N/S: 0.148 | N/S: 0.340 | N/S: 0.685 |
| >3 HLA-mismatches | N/S: 0.288 | N/S: 0.272 | N/S: 0.077 |
| **Table S2:** Univariate analyses of predictors of the secondary outcomes using binary logistic regression analysis. Given are odds ratio (OR) and the *p*-value of the likelihood-ratio test for selected variables. For non-selected (N/S) variable the *p*-value of the score test is displayed. | | | |

## Supplementary Table S3

| **Overall Recipient Survival (Cox Regression)** | **Model 1** | **Model 2** |
| --- | --- | --- |
| **Independent variable** | **HR (95% CI) *P*** | **HR (95% CI) *P*** |
| KDPI, % | N/S: 0.345 | - |
| Donor Age, years | - | N/S: 0.111 |
| Donor Sex (male) | N/S: 0.571 | N/S: 0.571 |
| Recipient Age, years | 1.037 (1.012 – 10.062) 0.004 | 1.037 (1.012 – 10.062) 0.004 |
| Recipient Sex (male) | N/S: 0.310 | N/S: 0.310 |
| Recipient BMI, kg/m² | N/S: 0.933 | N/S: 0.933 |
| Time on Dialysis, months | N/S: 0.056 | N/S: 0.056 |
| Cold Ischemia Time, hours | N/S: 0.885 | N/S: 0,885 |
| Previous RTx | N/S: 0.066 | N/S: 0.066 |
| Current PRA% | N/S: 0.776 | N/S: 0.776 |
| >3 HLA-mismatches | 0.518 (0.299 – 0.897) 0.019 | 0.518 (0.299 – 0.897) 0.019 |
| Donor Last Serum-Creatinine | - | N/S: 0.445 |
| Donor BMI, kg/m² | - | N/S: 0.667 |
| Donor Hypertension | - | N/S: 0.852 |
| Donor Diabetes mellitus | - | N/S: 0.621 |
| Donor Death by Anoxia | - | N/S: 0.245 |
| Donor cerebrovascular Death | - | N/S: 0.114 |
| Donor Death by Head Trauma | - | N/S: 0.518 |
| Donor Death by CNS-Tumor | - | N/S: 0.575 |
| Donor Death by other cause | - | N/S: 0.466 |
| **Table S3:** Multivariate analysis of predictors of overall recipient survival using stepwise forward selection in Cox regression analysis (for details, see Methods). Given are hazard ratios (HR) with 95% confidence interval (95% CI) and *p*-value of the likelihood-ratio test for selected variables. For non-selected (N/S) variable the *p*-value of the score test is displayed. | | |

## Supplementary Table S4

| **Primary Outcome 1-y-eGFR (Linear Regression)** | Regression coefficient B  (95%-confidence interval) | Level of significance *P* |
| --- | --- | --- |
| KDPI | -0.206 | < 0.001 |
| Donor Age | -0.268 | 0.004 |
| **Table S4:** Bivariate analysis of prediction of the 1-y-eGFR using linear regression analysis with KDPI and donor age both entered into the analysis without further covariates. Given are coefficients of regression (B) with 95% confidence interval (95% CI) and the *p*-value of the likelihood-ratio test. | | |

## Supplementary Table S5

| **ROC for death-censored allograft failure** | **AUC**  (Area under the [ROC] curve) | asymptotic **significance** | asymptotic 95% -  **confindence interval** |
| --- | --- | --- | --- |
| KDPI | 0.652 | < 0.001 | 0.573 – 0.730 |
| Donor Age | 0.621 | 0.003 | 0,547 – 0.709 |
| ECD | 0.578 | 0.071 | 0.495 – 0.661 |
| **Table S5:** Specific Parameters of the receiver operating characteristic (ROC) curves illustrating the impact of either KDPI, donor age and positive ECD-status on death censored allograft graft failure | | | |

## Supplementary Table S6

| **Outcome Parameter** | **SCD (n =306)** | **ECD (n = 274)** |
| --- | --- | --- |
| 1-y-eGFR, med. (IQR) | 54.5 (43.3 – 71.9) | 40.2 (31.1 – 51.8) |
| Acute Rejections, no (%) | 52 (17.0) | 46 (16.8) |
| DGF, no (%) | 72 (23.5) | 63 (23.0) |
| Surgical Complications, no (%) | 83 (27.1) | 93 (33.9) |
| TX-Failure, no (%) | 20 (6.5) | 30 (10.9) |
| Death, no (%) | 22 (7.2) | 38 (13.9) |
| **Table S6:** Descriptive analysis of SCD and ECD donor kidneys within the study cohort  (no = number; med. = median; IQR = interquartile range) | | |

## Supplementary Figure S1

**
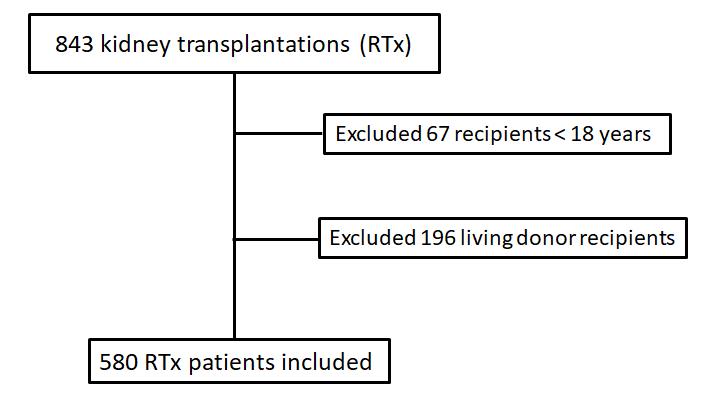
**

**Participant selection.** Flow chart illustrating participant selection: Between January 2007 and December 2014 843 Renal Transplantations have been conducted at the University Hospital Münster. Sixtyseven recipients younger than 18 years, and 196 adult living donor recipients have been excluded resulting in the study cohort of 580 consented RTX recipients.

# Supplementary Figure S2


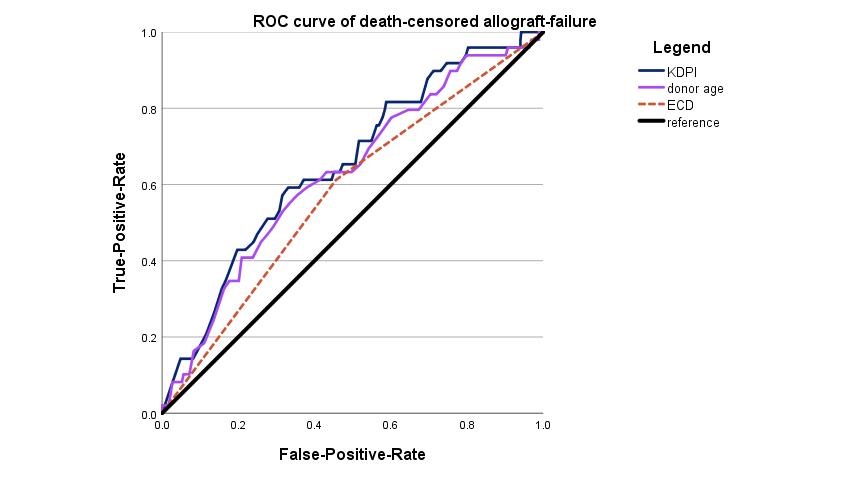


**ROC curves of KDPI, donor age and EDC-criterion.** Receiver operating characteristic (ROC) curves curves of KDPI, donor age (and ECD criterion) predicting death-censored allograft-survival (increasing age and KDPI were interpreted as gradually cumulating probabilities, positive ECD status as categorial prediction, of death-censored allograft failure, respectively implemented as state variable.): KDPI and donor age resulted in similar moderate but highly significant predictive values, whereas a true ECD-criterion does not significantly predict graft failure. (Table S4)
